# In search of mediators of leadership behavior to Team Creativity in Team Start-ups

**DOI:** 10.3389/fpsyg.2022.951603

**Published:** 2022-10-03

**Authors:** Tony Antonio, Agoes Tinus Lis Indrianto, Liestya Padmawidjaja

**Affiliations:** Universitas Ciputra, Surabaya, Indonesia

**Keywords:** Servant Leadership behavior, Transformational Leadership behavior, Team Ambidexterity, Team Climate, Team Creativity, Team Start-up

## Abstract

Creativity is believed as the first step to innovation, especially in a team or workgroup in an organization. Team Creativity will lead to several innovations in a team, such as product and process innovation. Team leaders play a significant role in embracing Team Creativity. Our study investigates the mediator variables to foster the impact of leadership behavior on Team Creativity in Team Start-up. Earlier research shows that two value-based leadership styles, Transformational and Servant Leadership, significantly affect a team's creativity. We proposed two mediators of leadership behavior to foster Team Creativity: Team Climate and Team Ambidexterity. The sample is early Team Start-ups in several cities in Indonesia, run and led by young people. It is empirical cross-sectional quantitative research with more than 434 participants aggregated into 145 teams. The result shows that Team Climate and Team Ambidexterity are good mediators of Servant and Transformational Leadership behavior to Team Creativity in Team Start-ups. The two variables maximize the impact of leadership behavior on Team Creativity.

## Introduction

Creativity as the generation of new and novel ideas is studied not only in the cognitive area of an individual or intrinsic personal motivation (Amabile, 1988; Woodman et al., 1993) but also as inter-personal collaboration/interaction within a team (Bullinger et al., 2004). Creativity is often emerging at the crossroads of divergent avenues of knowledge (Amabile and Conti, 1999) and inter-collaboration among individuals. In responding to the rapid change and the competitiveness in the business, a start-up relies on team creativity (Tjosvold et al., 2004), not individual creativity. More studies have been done to investigate the factors influencing Team Creativity (Shin and Zhou, 2007, p. 1,715; Shalley et al., 2009). Later studies have concluded that several aspects of the surrounding, such as leadership and organizational hierarchy, influence Team Creativity (Amabile et al., 2004; Artz et al., 2010).

Team Creativity has an essential effect on the success of an organization (Sun et al., 2016) and refers to the collective effort of every team member to create a new idea (Carmeli and Paulus, 2015). It evolves from a complex and contextual interaction among team members (Koh et al., 2019) and needs support and empowerment from the team leader (Zaccaro et al., 2001). Leadership behaviors are imperative for fostering Team Creativity in an organization or a team (Yang et al., 2017; Zhou et al., 2019). Investigating leadership behavior's role in Team Creativity and their mediators is vital for Team Start-ups since leadership behavior directs and influences creativity (Herrmann and Felfe, 2014) and develops competencies to encourage the process of creativity as well as opportunity recognition in the business (Swiercz and Lydon, 2002; Chen et al., 2009).

Earlier research shows that two value-based leadership styles, Transformational and Servant Leadership, significantly affect a team's creativity. Gumusluoglu and Ilsev (2009) study the influence of Transformational Leadership on creativity in an organization, followed by He et al. (2020), who investigate how Transformational Leadership facilitates individual creativity into team creativity. Yoshida et al. (2014) and Antonio et al. (2020) researched how Servant Leadership affects creativity in a team, while Chen et al. (2022) investigated the influence of Servant Leadership on creativity based on the Social Exchange Theory. The two-leadership style needs mediators to foster creativity in a team. The direct influence of leadership behaviors on Team Creativity needs to be empowered and maximized. We propose potential variables to mediate the influence.

Rosing et al. (2011) study the concept of Ambidexterity in team leadership and recommend that certain leadership behaviors are required to bring about the ambidexterity process of creativity and innovation. Jacob et al. (2015) studied the role of Ambidexterity at a team level and recommended investigating the potential antecedent to Team Ambidexterity that leads to creativity. Leadership behaviors are a good antecedent for Ambidexterity for creativity. We proposed two leadership behaviors—Servant Leadership and Transformational Leadership-to be investigated and Team Ambidexterity as the mediators for Team Creativity.

The basic understanding of climate in an organization was developed by Patterson et al. (2005) as an intervening variable between the organizational context and the member's behavior. Anderson et al. (2014) expand the idea of the climate in a work unit or team as a missing link between management and the team outcome. Team Climate is a means where team members could have information about the appropriate role behavior of the team members and the expected team outcomes. The expected outcome may vary depending on the characteristics of the team (Schneider et al., 2013). In a team context, a leader influences members through several paradoxical processes. Besides working on a dyadic basis to push the member to meet the performance demand, they also need to embrace a motivational climate and creative environment to bear team creativity (Zhang et al., 2021). Xu et al. (2019) recommend Team Climate as an antecedent to Team Creativity, while Team Climate is also positively related to and associated with positive leadership behavior (Piccolo and Colquitt, 2006; Shin and Zhou, 2007).

Finally, this study proposes two mediators of leadership behavior—Transformational Leadership and Servant Leadership behavior—to foster Team Creativity, namely Team Climate and Team Ambidexterity.

## Literature review and hypotheses development

### Team Starts-up

Early Entrepreneurship activities are primarily done in a team rather than in a lone ranger mode. For example, Ruef (2010) reports that almost 95% of the individuals starting a business either involve others or intend to collaborate later.

Forsström-Tuominen et al. (2017) found that this team-based entrepreneurship or Team Start-up is characterized by (i) the definition, (ii) the link between Team Start-up characteristics and team performance, and (iii) the antecedents and effects of team cognition. We will start by discussing some definitions of Team Start-up to find the base of start-up understanding and then explore the other two characteristics to build the theoretical model of this study.

Lazar et al. (2020) define Team Start-up as an entrepreneurial team that consists of individuals who have new business ideas and share ownership of the team, while Forsström-Tuominen et al. (2017) defined it as a team that consists of individuals who develop and establish a business with equity ownership, and commitment to common goals/outcomes. Bolzani et al. (2019) mention it as a group of individuals pursuing business opportunities. Every individual has a significant role and ownership interest in team management and directly influences the team's strategic choices.

Knight et al. (2020) expanded the research by providing a multidimensional conceptualization of the start-up framework with three key dimensions: first is the Ownership of Equity, second is the Autonomy of Strategic Decision-Making, and third is Entitavity. Ownership of Equity is the core dimension of a Team Start-up that explains the need, the amount, and the distribution of equity among team members. Autonomy of Strategic Decision-Making describes the exercising agency and the scope and authority of decision-making. Entitavity reflects the closeness of a team where the team is a unified whole, coherent, and unified organization entity. All the dynamics of the team range between these three dimensions, including Team Creativity and other inter-team interaction.

The theoretical framework of a Team Start-up is surveyed by Antonio et al. (2021) as follows: (i) “Theory of Entrepreneurship” of Cantillon (1775) and the “Creative Destruction” theory of Schumpeter (1942), which stated that a start-up is a combination of creativity, novelty, innovation, and development, (ii) the concept of Life Cycle Theory (Kaulio, 2003) which consider start-up as a linear and dynamic entity that address several challenges through several phases, and (iii) Complexity Theory (Tsai and Lan, 2006) which reveal that a start-up follows a stiff transition during the journey. This transition is called a threshold; in this case, using the threshold is the ultimate way to let a new order arise.

Considering the various definitions and the theoretical frameworks above, Team Start-up can be defined as an entrepreneurship entity consisting of two to three individuals committed to a common goal and identified by opportunity creation, creativity/innovation, and risk-taking.

### Team Creativity

Creativity is defined as the act of producing novel and purposeful ideas (West and Farr, 1990, p. 9). It is always associated with valuable and novel idea generations (Amabile, 1988, p. 126; Zhou and Shalley, 2010) and happens in specific periods (Woodman et al., 1993). Creativity is seen as the antecedent to innovation (Amabile and Conti, 1999; West, 2002; Klijn and Tomic, 2010). It occurs over the whole innovation implementation process (Tang, 2019). As part of the integral process of innovation, Anderson's integrative definition of creativity in the workplace is written as the integration of improved processes, outcomes, and products. The creative process has several stages, from idea generation to idea implementation. These stages aim for a better procedure, practice, or products (Anderson et al., 2014).

Creativity in a team is defined as a process of producing novel and purposeful ideas through several collaboration procedures among team members (Shin and Zhou, 2007, p. 1,715). Team Creativity is imperative to respond to the rapidly changing demand in the marketplace (Tjosvold et al., 2004).

Early theory to support Team Creativity is the Componential Theory (Amabile, 1997). The theory explains three major significant components of individual and Team Creativity. First, is the expertise of team leaders and team members, second is the thinking skill, and third is their intrinsic motivations. A later study by Amabile shows additional components to enhance employee creativity. They are motivated to innovate, providing resources and better managerial practices (Amabile and Conti, 1999).

Woodman et al. (1993) studied the Interactionist Theory of organizational creativity, which is considered one of the most developed theories on organizational creativity and innovation (Shalley et al., 2009; Yuan and Woodman, 2010; Zhou and Shalley, 2010). The theory explains the interaction process among individuals in a team and an organization which occurs in various stages of the institution, such as individual, team unit, and organization. Creativity is a result of holistic conditions of genetics, cognition, knowledge, social status, and surrounding contextual influence.

On the other hand, Team Creativity is composed of team members' creativity, team characteristics, team interaction, and the contextual impact on the team. Individual creativity and team creativity will initiate organizational creativity. From the Interactionist perspective, creativity is determined mainly by the interaction among aptitude, process, and environment to produce a novel and purposeful idea within a social context (Plucker et al., 2004).

Creativity depends on culture. Different cultures will determine various kinds of creativity (Anderson et al., 2014). At the individual level, culture will influence the process of how creativity emerges and the assessment method, while at the team level, culture will impose team creativity (Chiu and Kwan, 2010; Hempel and Sue-Chan, 2010).

Based on these theoretical frameworks, we define Team Creativity as the generation of new and purposeful ideas in a team through the interaction of working together among the team members.

### The mediator role of Team Ambidexterity

#### Team Ambidexterity

Ambidexterity combines exploration and exploitation to enhance creativity and innovation in team and organization performance (Raisch et al., 2009; Papachroni et al., 2015; Lee et al., 2017; Walrave et al., 2017; Luger et al., 2018). Bledow et al. (2009) laid out the ambidexterity theory and suggested that the exploration and exploitation activities should be engaged together to pursue creativity in a team or organization. This idea differs from the ambidexterity understanding proposed by Gupta et al. (2004), which mentioned that the two activities must be separated into two different activities. Rosing et al. (2011) echoed the idea of Bledow by proposing the integration of exploration and exploitation within the same system. Later research supports Bledow's ambidexterity theory (Zacher and Wilden, 2014; Zacher and Rosing, 2015; Zacher et al., 2016; Rosing and Zacher, 2017; Alghamdi, 2018; Klonek et al., 2020). The integration process of exploration and exploitation pursues the paradoxical demand to achieve creativity (Klonek et al., 2020). The mechanical process can be seen from the paradox perspective (Papachroni et al., 2015; Cunha et al., 2019). The integration of the paradox perspective and the ambidexterity theory lay an excellent framework to deal with the inherent complexity of an organization or team.

Team Ambidexterity consists of two key activities: team exploratory and team exploitation activities. Hammond and Farr (2011) and Rosing et al. (2011) used a dynamic model of workgroup theory for the operationalization of Team Ambidexterity which was proposed earlier by Farr et al. (2003).

Team exploratory is a set of supporting activities to reach creative outcomes during creativity (Rosing et al., 2011). This set of activities includes problem identification, potential solutions, and idea generation to optimize the opportunity. In addition, the team contributes multiple ideas on how to face the problem or opportunity (Girotra et al., 2010) and conceptual combination (Ward, 2004) and transformed into great creativity (Simonton, 2003). Through exploratory activities, teams will improve the success of the creative process during the creativity phase. Team exploitative activities refer to a series of activities facilitating the implementation of ideas during the creative process, which is based on the same dynamic model (Rosing et al., 2011). Therefore, it includes evaluating identified ideas in the creative phase and selecting ideas to be implemented. Evaluating the various ideas may lead to choosing the best idea based on the problem context, the creativity needs, and resource constraints (Hammond and Farr, 2011).

The performance of exploratory and exploitative activities in teams can be accomplished in several ways, namely engaging in paradoxical thinking (Gibson and Birkinshaw, 2004) and switching between exploratory and exploitative activities (Rosing et al., 2011). Previous research has also demonstrated that exploratory and exploitative activities can coincide within a team (Gilson et al., 2005; Kostopoulos and Bozionelos, 2011).

#### Team Ambidexterity and Team Creativity

Radomska and Wołczek (2020) analyzed 62 previous studies on the relationship between ambidexterity and creativity in an organization. Their finding is as follows: (i) there are four research perspectives on ambidexterity and creativity issue, namely learning process and knowledge acquiring, organizational context, managerial practice, and company's characteristic; (ii) creativity belongs to the managerial practice, which is a dominant perspective compared to the other three perspectives; (iii) to enhance creativity in an organization, we need to facilitate Team Ambidexterity to embrace the right approach.

Enhancing creativity is perceived as a challenge in finding the balance between the two aspects of Ambidexterity (Jones and Casulli, 2014; Radomska and Wołczek, 2020). However, finding this balance requires an ambidexterity-based approach (Lubatkin et al., 2006). Sheremata (2000) mentions the two aspects of Ambidexterity as a centrifugal and centripetal force in an organization. The two forces will foster the organization to act creatively and collectively to develop a new creative product.

*Hypothesis 1: Team Ambidexterity gives a positive impact on Team Creativity*.

#### Servant Leadership

Servant Leadership is a specific leadership type with a unique approach initiated by Greenleaf (1970). It is based upon characteristics such as Listening, Empathy, Persuasion, Conceptualization, Stewardship, Ethics, and an intention to serve others (Autry et al., 2001; Greenleaf, 2002; Blanchard and Hodges, 2003; Fisher, 2004). Larry Spears expands Greenleaf's initiation and highlights Servant Leadership as the new leadership model to serve and prioritize followers' needs (Spears, 1996). Spear's concept of Servant Leadership focuses on the holistic aspects of leadership in the workplace and community. It introduces the principle of power-sharing in decision-making.

Recent research mentions Servant Leadership as holistic and multi-dimensional leadership that covers the leaders' and followers' rational, relational, ethical, emotional, and spiritual aspects (Sendjaya and Cooper, 2011). The comprehensive approach enables leaders to completely address those dimensions that cannot be found in other leadership approaches (Barbuto and Wheeler, 2006; Liden et al., 2008). Sendjaya has three points to explain. First, servant leadership reflects a hearty internal orientation to serve others. Second, it is a follower-centered approach to leadership. Third, it is a holistic approach where leaders emphasize seeking the positive difference of the followers (Sendjaya et al., 2008). Finally, it will create a multi-aspect engagement between leaders and followers, which empowers the followers to grow to their best performance (Eva et al., 2019).

As stated in Eva et al. (2019), Servant Leadership is built on several conceptual frameworks, such as the Power theory (French et al., 1959), Social Exchange Theory (Blau, 1964), Social Learning Theory (Bandura and Walters, 1977), Social Identity Theory (Tajfel, 1978), and Conservation of Resource (Hobfoll, 1989).

Power Theory is a useful theoretical framework to explain the influence of a servant leader (Sikorski, 2016) and describes how leaders exercise their impact on their followers. The theory was established by French et al. (1959) and expanded by Baron-Cohen (1999). Some of the powers are reward, coercive, and legitimate. Reward power is the ability of a leader to give a reward, coercive power is the ability of a leader to punish (Sikorski, 2016), and legitimate power is the ability of a leader to influence subordinates. The Social Exchange Theory (SET; Blau, 1964) explains the relationship between servant leaders and their followers since SET is based on the norm of reciprocity. Social Learning Theory (Bandura and Walters, 1977) explains that leaders are role models in attitude, value, and behavior. Servant leaders are viewed as role models as they act altruistically to serve others (Schwarz et al., 2016). Social Learning Theory describes how the leaders influence the performance of the followers through modeling (Liden et al., 2014) and encourages creativity and innovation for the followers (Newman et al., 2017). Social Identity Theory (Tajfel, 1978) explains why and how servant leaders consider the followers as partners in the organization/team through empowering followers' identification (Chunghtai, 2016), prototyping leader identification (Yoshida et al., 2014), and Team Climate (Chen et al., 2015). These social theories help us understand servant leaders' behavior that makes them different from other types of leaders.

Servant Leadership can be defined as a holistic leadership approach that influences the follower by focusing more to serve the followers not only for the organization's objective but also on developing the full potential of the followers. Servant leaders do understand that by focusing on the followers there will be an increase in several critical issues such as productivity, teamwork, and customer service.

#### Servant Leadership and Team Ambidexterity

When leaders stimulate the mind of their followers, this will encourage them to not stay with how things are and think beyond what is comfortable. However, it might also positively change their qualitative creativity and cognitive conflict (De Dreu, 2006).

Ambidexterity follows a non-linear, complex, and complicated process. In balancing this complex interaction, particular leadership behavior is needed. Rosing expanded the concept of ambidexterity of leadership to team creativity (Rosing et al., 2011). Thus, specific leadership behavior is required to manage the ambidexterity process. Bledow et al. (2009) argue that the current leadership style cannot integrate the leadership behavior needed to accommodate the exploitation and exploration process, while Gupta et al. (2004) believe that the most critical leadership feature for creativity is the development of exploration by increasing the variant of each follower's behavior. Moreover, Chang and Hughes (2012) reported that leadership behavior for ambidexterity is marked by the ability to adapt and the courage to take a risk.

Servant leaders are genuinely focused on the development of their followers (Hu and Liden, 2011; Van Dierendonck, 2011), and it displays an altruistic commitment to helping followers to grow. Following the work of Yoshida et al. (2014), where Servant leadership directly influences affect-based trust rather than cognitive-based within the team, and the study of Antonio et al. (2021) on the impact of Servant Leadership on Team Ambidexterity, we hypothesize that:

*Hypothesis 2: Servant Leadership behavior gives a positive impact on Team Ambidexterity*.

#### Transformational Leadership

James MacGregor Burns gives a basic understanding of Transformational Leadership as a mutual collaboration between leaders and followers in helping each other to advance to a higher level of morale and motivation for the benefit of the team, organization, or community (Burn, 1978). Bernard M. Bass developed a more comprehensive definition from the psychological mechanism perspective and explained how the transformation process of a follower happens through four dimensions: individual consideration, intellectual stimulation, inspirational motivation, and idealized influence (Bass and Bass, 2009).

Individualized consideration is the condition where the leader listens to each follower's needs and gives mentoring and coaching. They treat followers as individuals by identifying their different needs, knowing each follower's ability, and respecting their aspirations (Braun et al., 2013). With intellectual stimulation, leaders encourage and motivate their followers through cognitive stimulation. They nurture and develop people to think independently, challenge assumptions, take risks, and solicit followers' ideas. These two dimensions of Transformational Leadership—individual consideration and Intellectual stimulation—stimulate the exploration by enhancing team members' self-esteem, supporting their individual needs, and encouraging them to convey their opinions (Nemanich and Vera, 2009).

Inspirational Motivation is where leaders challenge a higher standard of achievement, share the goals, and pass the optimism to the followers. Dimas et al. (2018) studied how Social Cognitive Theory supports the self-efficacy of the follower led by a transformational leader. The last dimension, Idealized Influence, is where leader exercise their influence as role models to provide for high ethical behavior and gain respect and trust from the followers (Bass and Bass, 2009). They share their knowledge and ideas to facilitate cooperative and efficient working among their followers (Aryee et al., 2012). Inspirational motivation and idealized influence are associated with inclusive and supportive behavior, which makes Transformational Leadership can exploit collective self-construal and self-efficacy (Elenkov and Manev, 2005). Transformational leadership correlates to the critical processes of Ambidexterity in the exploitation and dissemination of the team knowledge and information reservation (Amitay et al., 2006). The exploration and exploitation effects of the four dimensions of Transformational Leadership become the important driver for the exploration and exploitation of a team (Jansen et al., 2006). It will lead to:

*Hypothesis 3: Transformational Leadership gives a positive impact on Team Ambidexterity*.

With hypotheses 1, 2, and 3, we propose Team Ambidexterity as the mediator between leadership behaviors and Team Creativity.

### The mediator role of Team Climate

#### Team Climate

The climate in a team may be defined as a means where team members derive information about their expected and appropriate role behavior to attain the team outcomes (Schneider et al., 2013). It examines the team members' perceptions and experiences of embracing the work group's creative endeavors (Hunter et al., 2007). Team Climate will create shared perceptions of team members regarding the team policies, team procedures, and functional interaction in the team (Zohar and Tenne-Gazit, 2008) and construct a creative process where creative behavior leads to creative solutions (Anderson et al., 2014) concerning developing creative sourcing strategy of the team (Kiratli et al., 2015). Liang et al. (2010) studied the significant impact of Team Climate on the team members' perceptions and beliefs.

Further research shows that Team Climate differs between teams because of team-specific differences rather than organization-wide differences (Ashkanasy and Nicholson, 2003; Herman et al., 2008). Furthermore, it shows that the share of perception of effect at the team level is more significant than at the organizational level.

Based on different theoretical frameworks, several Team Climates models have been developed based on several concepts; such as (i) the West model, which is based on the theory of motivation (West, 1990); (ii) the Amabile model, which is rooted in intrinsic motivation theory and focused on the more considerable organizational climate (Amabile and Conti, 1999); (iii) the Ekval model, which focused on integrating several dimensions of psychological processes theory (Ekvall, 1996) and (iv) the three-dimension model of affiliation, trust, and innovation, which is based on social influence and social behavior stated by Bock et al. (2005).

The first four-factor model of Team Climate is proposed by West and Farr (1990) and then expanded by West and Anderson (1996) and improved by Anderson et al. (2014). The four aspects of the four-factor model are vision, participative safety, task orientation, and support for innovation. Vision is defined as a valued outcome that represents a higher-order goal and a motivation vigor at work. It embodies clarity, visionary nature, attainability, and sharedness. Safety participation reveals the safety of the team member when they are implicated in the decision-making process. It relates to the active involvement of the team member, trustworthiness among members, leader support, and mostly not feeling threatened. The task orientation describes a general commitment to excellence in task performance in connection with the shared vision. Creativity support is the expectation, approval, and support to improve the fresh ideas of doing things at work. The support level may differ among teams (Anderson et al., 2014).

#### Team Climate and Team Creativity

A Team Climate for creativity accommodates a team's values and norms to emphasize creativity and innovation (West and Anderson, 1996). Creativity climate is considered a method in which the negative effect of work demands on organizational performance may be improved (King et al., 2007). In a supportive situation, team members will be triggered to develop new approaches, explore potential solutions, and attempt to practice new problem-solving activities (Baer and Oldham, 2006). The challenge to the supporting climate will come when the team puts more on efficiency and reliability than the performance outcomes (Hirst et al., 2009). Team Climate is needed to accommodate and influence the relationship between the creative process and company performance (Baer and Frese, 2003).

*Hypothesis 4: Team Climate gives a positive impact on Team Creativity*.

#### Team Climate and leadership behavior

The climate in an organization also plays an intervening variable in employee behavior (Patterson et al., 2005). A work unit or team mediates the gap between management and expected outcomes (Anderson et al., 2014). The expected outcome may vary depending on the context and the level difference of the organization (Schneider et al., 2013). Some examples of the outcome are creative performance (Si and Wei, 2012), firm performance (Baer and Frese, 2003), safety (Zohar and Tenne-Gazit, 2008), and innovation (Antonio et al., 2021).

Kinnunen et al. (2016) report a study on the relationship between Leadership and Team Climate. While, Liu et al. (2012) conclude their research that team leaders empowering behavior will increase the Team Climate, Xue et al. (2011) reported that the influence of empowering leadership behavior on extrinsic and extrinsic motivation is not the same. A leader with extrinsic motivation will provide guidance and fair treatment to team members and respect their input for the team's sake. Recent research by Coffeng et al. (2021) mentions that Empowering leadership influences the Team Climate for joint decision-making.

We propose two types of leadership to investigate the impact of leadership behavior on Team Climate, i.e., Servant leadership as a horizontal leadership and transformational leadership as a vertical type of leadership.

*Hypothesis 5: Servant Leadership gives a positive impact on Team Climate*.
*Hypothesis 6: Transformation Leadership gives a positive impact on Team Climate*.

With hypotheses 4, 5, and 6, we propose Team Climate as the mediator between leadership behaviors and Team Creativity.

## Materials and methods

### Research model

**Figure 1:**
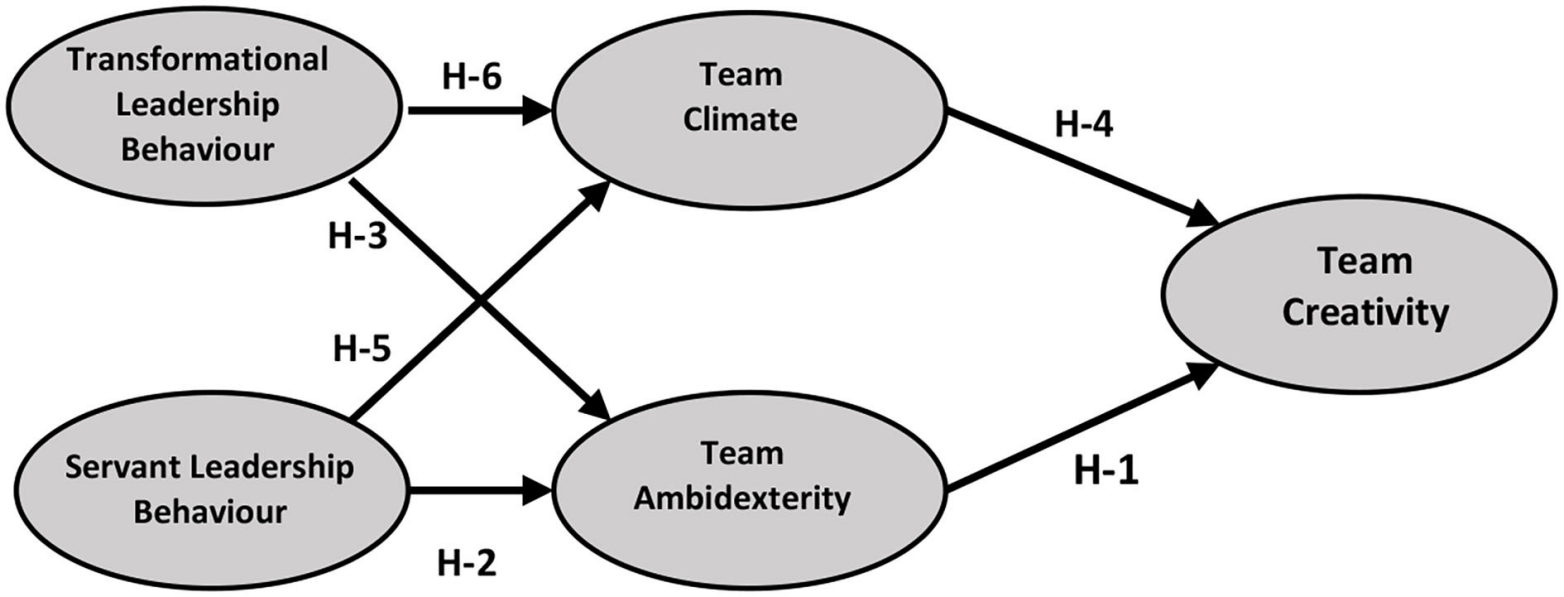
The theoretical model.

We design a theoretical model from the proposed hypotheses as illustrated in Figure 1. It has five variables that make Team Creativity the dependent variable with two mediator variables i.e., Team Ambidexterity and Team Climate, and two of the independent variables are Servant Leadership behavior and Transformational Leadership behavior.

### Research method

This study used early start-up teams in Indonesia as the population. The early start-ups in several cities in Indonesia, such as Jakarta, Bandung, and Surabaya, are chosen as the unit of analysis. Thus, different types of start-ups will also be examined, such as government-sponsored, private initiatives, and university-based start-ups. The elected start-ups should have a minimum of 1 year of operation to ensure the team has experienced some innovation journeys during the business activities.

A total of 434 purposive non-probability samples were involved in this research. The samples were then grouped into 145 teams. The member perceptions of the leader are the focus of this study. The measurement instrument is being circulated to the member of the start-up in digital form (Google Forms and email) as a survey questionnaire. The survey questions are translated into Bahasa Indonesia (Indonesian) to make it easier for the respondents. The questionnaire questions are grouped into five categories of variables to avoid common method variance. This empirical study uses quantitative data analysis using version 3.2.9 of the Smart Partial Least Square (PLS) procedure (Hair et al., 2019). The reliability and validity of the outer model are analyzed, while the structural model assessment encompasses the coefficient of determination and the study's hypothesis.

### Measurement instrument

Measures of the variables are outlined below. Detailed items of the measurement are given in the Appendix. The measurement uses the scale with five options ranging from 1, “not at all characteristics,” to 5, “very characteristic.”

Team Creativity is measured using a scale developed by Zhou and George (2001), an updated version of the Scott and Bruce (1994) measurement scale. There are 12 questions in the Zhou measurement scale to accommodate the creativity dimensions. The Servant Leadership scale is the Servant Leadership Behavior Scale (SLBS) which is developed by Sendjaya et al. (2019). There are six questions included in SLBS. The scale has been used in both Western (Australia) and Eastern (Indonesia) contexts (Sendjaya and Pekerti, 2010; Sendjaya and Cooper, 2011), specifically in business entities. Transformational Leadership behavior is measured using the Multifactor Leadership Questionnaire (MLQ) developed by Avolio and Bass (1995). It has seven questions about the four dimensions of Transformational leadership. Accessing open and closed leadership behavior is the way to measure ambidexterity. The tool to measure was developed by Rosing et al. (2011) and expanded by Zacher and Rosing (2015). The elements include monitoring and controlling goal attainment, controlling adherence to rules, taking corrective action, and paying attention to uniform task accomplishment. They also include diverse ways of finishing a task, encouraging experimentation within, giving room for ideas, and encouraging error in learning. Anderson and West (1996) developed Team Climate Inventory (TCI) based on West's work in 1996. Thus, a shorter version of TCI was developed by Kivimaki and Elovaino with only 14 questions to answer (Kivimaki and Elovainio, 1999). The indicators used are the attitude toward team objectives, which make the member feel understood and accepted. Information is shared within the team, allowing the team to be open, and they appraise weaknesses to achieve an outcome and give time to develop creative ideas.

### Results and data analyses

Table 1 reveals the size and profile of each start-up used as a sample. The demography of the samples is as follows: (i) The members of each start-up range from 1 to 5 people. (ii) The members are below 30 years old and hold an academic degree from graduate diplomas up to doctoral qualifications, which consists of various academic disciplines. (iii) All Start-up has been at least 1 year of operation; only a few have lasted more than 2 years. (iv) Team Start-ups come from several types of business areas, as shown in Table 1.

**Table 1:** Sample profile.

**Item**	**Segment**	**Frequency**	**Percentage**
Gender	Male	265	61.0
	Female	169	39.0
Age	Student	92	21.2
	Vocational	35	8.1
	Uni grad	289	66.6
	Master's degree	17	3.9
	Doctoral degree	1	0.2
Business	Tourism/culinary	43	29.7
	Personal dev	22	15.2
	Design	16	11.0
	Trading	16	11.0
	Technology	8	5.5

The result analysis refers to the PLS method by Hair et al. (2019). The reflective measurement model assessment covers the outer and inner evaluation. The evaluation includes convergent validity, discriminant validity, and composite reliability, then discuss the *R*-square, internal consistency reliability assessment, and path analysis.

#### Convergent validity

**Figure 2:**
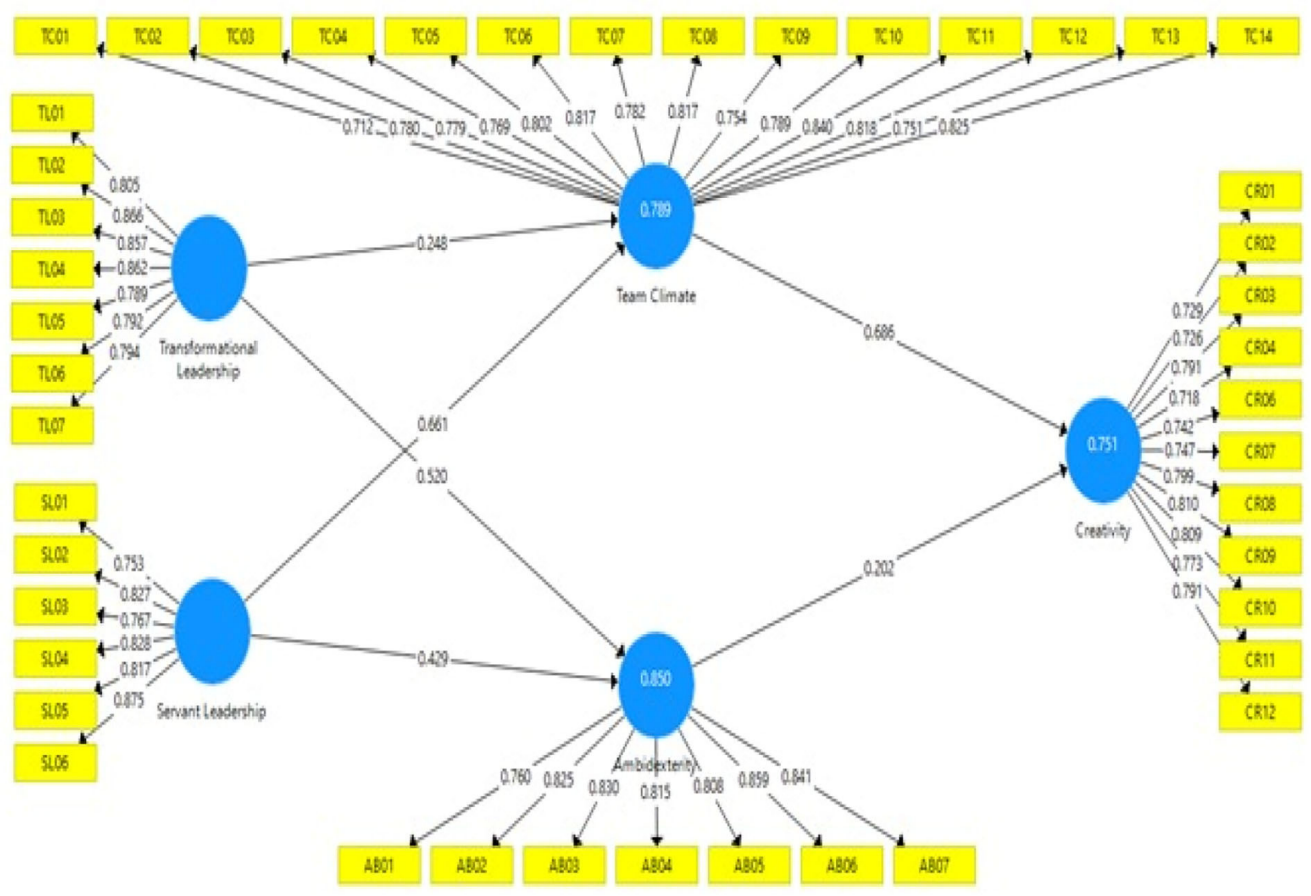
Research empirical model.

The result of the analyses is shown in Figure 2 and Table 2. Figure 2 shows that the value of the loading factor is >0.7, which means the indicator is valid for measuring its construction. All the average variance extracted (AVE) values displayed in Table 2 are higher than 0.5, which satisfies the requirement of convergent validity.

**Table 2:** Outer loading, AVE, and *t*-statistic.

**Variable**	**Indicator**	**Outer model**	**AVE**	***T*-statistics**
Ambidexterity	AB01	0.760	0.673	17.008
	AB02	0.825		25.675
	AB03	0.830		26.283
	AB04	0.815		23.560
	AB05	0.808		21.539
	AB06	0.859		33.976
	AB07	0.841		28.031
Creativity	CR01	0.729	0.589	14.583
	CR02	0.726		12.443
	CR03	0.791		20.664
	CR04	0.718		13.863
	CR06	0.742		14.205
	CR07	0.747		16.907
	CR08	0.799		24.573
	CR09	0.810		27.284
	CR10	0.809		20.909
	CR11	0.773		18.807
	CR12	0.791		20.398
Servant Leadership	SL01	0.753	0.660	15.126
	SL02	0.827		24.576
	SL03	0.767		11.286
	SL04	0.828		24.344
	SL05	0.817		23.887
	SL06	0.875		41.531
Team Climate	TC01	0.712	0.622	13.777
	TC02	0.780		18.947
	TC03	0.779		19.025
	TC04	0.769		19.079
	TC05	0.802		20.983
	TC06	0.817		24.737
	TC07	0.782		19.715
	TC08	0.817		24.145
	TC09	0.754		18.265
	TC10	0.789		21.884
	TC11	0.840		28.812
	TC12	0.818		26.556
	TC13	0.751		17.928
	TC14	0.825		28.786
Transformational Leadership	TL01	0.805	0.680	18.222
	TL02	0.866		31.029
	TL03	0.857		26.739
	TL04	0.862		33.913
	TL05	0.789		17.523
	TL06	0.792		22.428
	TL07	0.794		19.627

#### Discriminant validity

Two kinds of tests are used for Discriminant validity. First is the Fornell-Larcker criteria, where the AVE value must be higher than the *R*^2^ in all other latent variables. The second criteria are the cross-loading indicators which must be a higher correlation with other latent variables than their own. Based on the result in Table 3 (cross-loading) and Table 4 (correlation between variables), both the AVE and the cross-loading meet the criteria. Based on the two tables, it can be concluded that the Discriminant Validity assessment is valid.

**Table 3:** Cross loading.

**Indicator**	**Ambidexterity**	**Creativity**	**Servant Leadership**	**Team Climate**	**Transformational Leadership**
AB01	**0.760**	0.598	0.683	0.657	0.641
AB02	**0.825**	0.646	0.734	0.708	0.718
AB03	**0.830**	0.641	0.772	0.722	0.790
AB04	**0.815**	0.670	0.728	0.715	0.791
AB05	**0.808**	0.655	0.689	0.709	0.683
AB06	**0.859**	0.700	0.734	0.723	0.758
AB07	**0.841**	0.665	0.768	0.752	0.776
CR01	0.559	**0.729**	0.601	0.623	0.577
CR02	0.584	**0.726**	0.568	0.590	0.573
CR03	0.624	**0.791**	0.584	0.673	0.570
CR04	0.489	**0.718**	0.534	0.606	0.565
CR06	0.549	**0.742**	0.561	0.634	0.579
CR07	0.596	**0.747**	0.573	0.588	0.570
CR08	0.604	**0.799**	0.634	0.704	0.598
CR09	0.678	**0.810**	0.672	0.730	0.678
CR10	0.656	**0.809**	0.638	0.717	0.662
CR11	0.654	**0.773**	0.604	0.696	0.648
CR12	0.710	**0.791**	0.646	0.681	0.644
SL01	0.647	0.547	**0.753**	0.632	0.616
SL02	0.743	0.712	**0.827**	0.783	0.748
SL03	0.669	0.571	**0.767**	0.707	0.692
SL04	0.755	0.625	**0.828**	0.700	0.722
SL05	0.735	0.664	**0.817**	0.707	0.739
SL06	0.782	0.694	**0.875**	0.755	0.802
TC01	0.616	0.623	0.681	**0.712**	0.603
TC02	0.638	0.672	0.606	**0.780**	0.599
TC03	0.735	0.624	0.738	**0.779**	0.672
TC04	0.701	0.714	0.724	**0.769**	0.699
TC05	0.665	0.672	0.649	**0.802**	0.613
TC06	0.650	0.661	0.653	**0.817**	0.623
TC07	0.601	0.693	0.620	**0.782**	0.589
TC08	0.649	0.663	0.660	**0.817**	0.635
TC09	0.718	0.595	0.686	**0.754**	0.662
TC10	0.704	0.695	0.689	**0.789**	0.677
TC11	0.721	0.722	0.760	**0.840**	0.718
TC12	0.803	0.741	0.765	**0.818**	0.750
TC13	0.661	0.694	0.724	**0.751**	0.649
TC14	0.709	0.715	0.745	**0.825**	0.704
TL01	0.689	0.613	0.687	0.634	**0.805**
TL02	0.758	0.692	0.743	0.714	**0.866**
TL03	0.750	0.650	0.744	0.701	**0.857**
TL04	0.803	0.696	0.770	0.724	**0.862**
TL05	0.704	0.619	0.752	0.654	**0.789**
TL06	0.749	0.599	0.735	0.684	**0.792**
TL07	0.736	0.691	0.697	0.701	**0.794**

**Table 4:** The root of AVE and correlation between variables.

**Variable**	**AVE**	**Root AVE**	**Correlation between variables**				
			**Ambidexterity**	**Creativity**	**Servant Leadership**	**Team Climate**	**Transformational Leadership**
Ambidexterity	0.673	0.820	1				
Creativity	0.589	0.767	0.797	1			
Servant Leadership	0.660	0.812	0.890	0.785	1		
Team Climate	0.622	0.788	0.869	0.861	0.881	1	
Transformational Leadership	0.680	0.824	0.901	0.791	0.889	0.835	1

#### Reliability assessment

To evaluate the reliability, we evaluate the value of Cronbach's alpha and the value of composite reliability. Table 5 shows that all Cronbach's alpha is ≥0.7 and all Composite Reliability is ≥0.7 as well. These results meet the criteria of the Internal Consistent reliability assessment. The constructs are reliable.

**Table 5:** Cronbach's alpha and composite reliability.

**Variable**	**Cronbach's alpha**	**Composite reliability**
Ambidexterity	0.919	0.935
Creativity	0.930	0.940
Servant Leadership	0.896	0.912
Team Climate	0.953	0.958
Transformational Leadership	0.921	0.937

#### Influence of exogenous latent variable assessment

The *R*^2^ is defined as the magnitude of the variability of endogenous variables that able to be explained by exogenous variables. Chin (1998) recommended three classifications of *R*^2^: the first classification is substantial for *R*^2^ ≥ 0.67, the second classification is moderate for *R*^2^ ≥ 0.33 and the last is a weak classification for *R*^2^ ≥ 0.19. Table 6 shows all variables have >0.67 in *R*^2^, which belong to the substantial category.

**Table 6:** The *R*-square.

	***R*-square**
Ambidexterity	0.850
Creativity	0.751
Servant Leadership	0.789

#### Predictive relevance assessment

The Predictive Relevance assessment is executed by calculating the (Q2) value. The research model considers a relevance prediction for the Q2-value close to 1 (Hair et al., 2019). Using the formulation of Q2 as follows: Q2 = 1- (1-R12) (1 – R22), where R12 and R22 are the R-square of the endogen variable (Team Ambidexterity and Team Climate). Substituting the value gives a Q2-value of 88%. The value is more than 0, indicating an excellent exogenous latent variable (corresponding) as an explanatory variable and foreseeing its endogenic variables.

#### Hypothesis evaluation

The performance of the inner model is assessed using bootstrap resampling procedures. A bootstrap resampling procedure can evaluate it. The result is tabulated in Tables 7, 8. As shown in the table, the *T*-statistics value (higher than 1.96) and the *p*-value (<0.05) mean that all the indicator variables used are significant and all hypotheses are supported.

**Table 7:** Outer loading and *t*-statistic.

	**Original sample (O)**	**Sample mean (M)**	**Standard dev (STDEV)**	***T*-statistics (|O/STDEV|)**	***P*-values**
AB01 <- Ambidexterity	0.760	0.756	0.046	16.375	0.000
AB02 <- Ambidexterity	0.825	0.820	0.033	24.650	0.000
AB03 <- Ambidexterity	0.830	0.828	0.032	26.184	0.000
AB04 <- Ambidexterity	0.815	0.812	0.035	23.159	0.000
AB05 <- Ambidexterity	0.808	0.806	0.040	20.450	0.000
AB06 <- Ambidexterity	0.859	0.856	0.027	31.391	0.000
AB07 <- Ambidexterity	0.841	0.841	0.032	26.453	0.000
CR01 <- Creativity	0.729	0.724	0.048	15.277	0.000
CR02 <- Creativity	0.726	0.718	0.056	12.889	0.000
CR03 <- Creativity	0.791	0.787	0.039	20.251	0.000
CR04 <- Creativity	0.718	0.716	0.049	14.605	0.000
CR06 <- Creativity	0.742	0.736	0.054	13.660	0.000
CR07 <- Creativity	0.747	0.746	0.043	17.329	0.000
CR08 <- Creativity	0.799	0.800	0.034	23.707	0.000
CR09 <- Creativity	0.810	0.812	0.029	28.324	0.000
CR10 <- Creativity	0.809	0.808	0.038	21.380	0.000
CR11 <- Creativity	0.773	0.770	0.044	17.569	0.000
CR12 <- Creativity	0.791	0.790	0.040	19.955	0.000
SL01 <- Servant Leadership	0.753	0.746	0.054	13.940	0.000
SL02 <- Servant Leadership	0.827	0.825	0.039	21.147	0.000
SL03 <- Servant Leadership	0.767	0.764	0.072	10.700	0.000
SL04 <- Servant Leadership	0.828	0.832	0.033	25.112	0.000
SL05 <- Servant Leadership	0.817	0.819	0.033	25.118	0.000
SL06 <- Servant Leadership	0.875	0.874	0.023	38.881	0.000
TC01 <- Team Climate	0.712	0.708	0.050	14.161	0.000
TC02 <- Team Climate	0.780	0.775	0.043	18.059	0.000
TC03 <- Team Climate	0.779	0.778	0.038	20.618	0.000
TC04 <- Team Climate	0.769	0.766	0.039	19.682	0.000
TC05 <- Team Climate	0.802	0.798	0.037	21.761	0.000
TC06 <- Team Climate	0.817	0.814	0.035	23.452	0.000
TC07 <- Team Climate	0.782	0.778	0.038	20.834	0.000
TC08 <- Team Climate	0.817	0.815	0.035	23.281	0.000
TC09 <- Team Climate	0.754	0.751	0.043	17.406	0.000
TC10 <- Team Climate	0.789	0.784	0.037	21.252	0.000
TC11 <- Team Climate	0.840	0.839	0.031	27.070	0.000
TC12 <- Team Climate	0.818	0.817	0.032	25.340	0.000
TC13 <- Team Climate	0.751	0.747	0.045	16.808	0.000
TC14 <- Team Climate	0.825	0.824	0.030	27.569	0.000
TL01 <- Transformational Leadership	0.805	0.799	0.049	16.501	0.000
TL02 <- Transformational Leadership	0.866	0.864	0.029	29.830	0.000
TL03 <- Transformational Leadership	0.857	0.856	0.030	28.190	0.000
TL04 <- Transformational Leadership	0.862	0.860	0.026	33.702	0.000
TL05 <- Transformational Leadership	0.789	0.786	0.048	16.291	0.000
TL06 <- Transformational Leadership	0.792	0.792	0.038	21.079	0.000
TL07 <- Transformational Leadership	0.794	0.790	0.039	20.250	0.000

**Table 8:** Path coefficient and *t*-statistic.

	**Original sample (O)**	**Sample mean (M)**	**Standard dev (STDEV)**	***T*-statistics (|O/STDEV|)**	***P*-values**
Team Ambidexterity—Team Creativity	0.202	0.212	0.106	1.896	0.059^*^
Servant Leadership—Team Ambidexterity	0.429	0.429	0.095	4.527	0.000^**^
Servant Leadership—Team Climate	0.661	0.679	0.107	6.204	0.000^**^
Team Climate—Team Creativity	0.686	0.676	0.102	6.722	0.000^**^
Transformational Leadership -> Team Ambidexterity	0.520	0.518	0.093	5.607	0.000^**^
Transformational Leadership -> Team Climate	0.248	0.230	0.114	2.164	0.031^**^

(*) indicates the p value of 0.059 which is higher than 0.05 (accuracy 94,1%, lower than 95%).

(**) indicates the p value 0.000 (accuracy higher than 95 %).

To summarize the structural model assessment, it shows that the value of *R*^2^, predictive relevance, and the relationship between variables are satisfactory since both the outer and inner model meets the standard. The assessment also shows that all hypothesis is supported.

#### Mediation analysis

**Figure 3:**
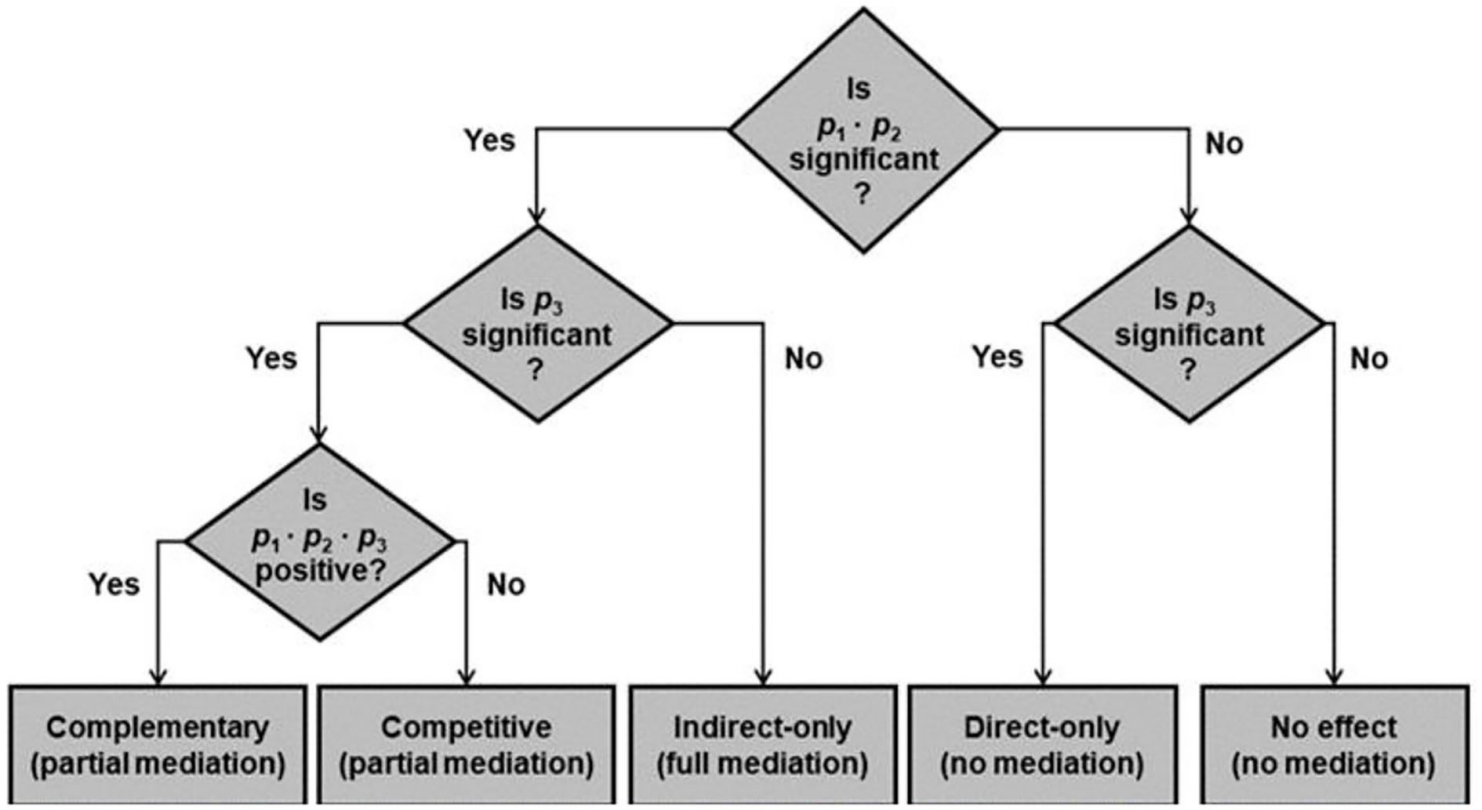
Decision tree of analyzing the mediation effect (Zhao et al., 2010).

Zhao et al. (2010) presented a conceptual method of mediation analysis that is echoed by other researchers (Nitzl et al., 2016; Hair et al., 2017; Memon et al., 2018). Zhao et al. proposed five mediation kinds: (i) Direct-only mediation, (ii) No-effect mediation, (iii) Indirect-only mediation, (iv) Competitive mediation, and (v) Complementary mediation. The concept is plotted into a flowchart in Figure 3. The *p*-value among the mediator variable in Figure 2 can be summarized in Table 9. Substituting these significant *p*-values to the flowchart will give us the result that both mediator variables are partial complementary mediation.

**Table 9:** *p*-value among variables.

	**Code**	**Value**	**Significant**
Team Ambidexterity—Team Creativity	p_1_	0.202	Significant
Servant Leadership behavior—Team Ambidexterity	p_2_	0.429	Significant
Transformational Leadership—Team Ambidexterity	p_3_	0.520	Significant
Team Climate—Team Creativity	P_4_	0.686	Significant
Servant Leadership behavior—Team Climate	p_5_	0.661	Significant
Transformational Leadership—Team Climate	p_6_	0.248	Significant

## Discussion

This study aims to give a systematic, evidence-based mediation effect between leadership behaviors and Team Creativity in Team Start-ups. The mediation analyses support the fact that the mediation effect of the two mediators is partial complementary mediation which means that the mediation effect exists with the direct effect pointing in the same direction (Zhou and Shalley, 2010).

### The mediating role of Team Climate and Team Ambidexterity

Both mediators have a direct effect on Team creativity and mediate leadership behaviors. The mediator role of Team Climate is significant while the impact of Team Ambidexterity is considered weak. Early research reports that Climate only moderates creativity (Eisenbeiss et al., 2008) and impacts creativity on the personal level (Xue et al., 2011; Xu et al., 2019). Our finding confirms that Team Climate is a good mediator and impacts the creativity of the team. The correlation value between Team Climate and Team Creativity indicates that Team Climate influences Team Creativity more than Team Ambidexterity. Servant Leadership is also an excellent antecedent to Team Climate compared to Transformational Leadership, with a correlation value of 0.661. The result leads to the point that Team Climate is a good mediator between Servant Leadership and Team Creativity.

Our study reveals that the impact of Team Climate is three times higher compared to Team Ambidexterity. While earlier studies by Jacob et al. (2015) and Antonio et al. (2020) show that Ambidexterity is a good antecedent to creativity in a team, our finding expands the idea that as the mediator, Team Ambidexterity gives a weak impact.

### Leadership behavior and Team Creativity

Leaders should stimulate their follower's creativity (Bledow et al., 2009), but the mechanism of the simulation process still needs more exploration. Our study tries to answer the question that Burke et al. (2006) asked on the leadership behavior that can function well in a team. The study shows that both Transformational Leadership behavior and Servant Leadership behavior can function well in teams with proper mediators. Our finding echoes the work of Cengiz Ucar et al. (2021), who report that Servant Leadership and Transformational Leadership directly affect team member creativity. The correlation between the research variables shows that Servant Leadership is a better antecedent to Team Climate and Team Creativity. At the same time, Transformational Leadership is better for Team Ambidexterity which opens a question on the different leadership behavior between vertical and horizontal leadership styles.

#### Theoretical contribution

This study extends the leadership theory and the ambidexterity theory in the context of a start-up team. The two leadership styles, Transformational Leadership and Servant Leadership which are mostly applied in the organizational or company context can be implemented in the Team Start-up context. This extension is important for the leadership theory because it underlies the role of leadership in a start-up team. Servant Leadership which has more shared authority among the member give more impact on Team Creativity compared to the vertical approach of Transformational Leadership. Both leadership approaches work well in Indonesian culture.

The other theory contribution is the role of Servant Leadership as the antecedent to Ambidexterity. This combination of motivational-based and process-based leadership give a higher impact on team creativity or team performance at large. Servant Leadership is also a good antecedent to Team Climate (behavioral-based theory of West) which in turn influences creativity.

The extent of ambidexterity theory shows that the exploration and exploitation processes give less impact on the team creativity compared to the two leadership approaches. It indicates that early Team Start-ups need a more guided or motivational leadership style rather than process based. Combining different streams of research advances our understanding of the relationship between leadership, processes, and creativity.

#### Practical implications

Since the research shows that Team Climate is a good mediator of leadership behavior on Team Creativity. Intentionally, all start-ups need to build a conducive atmosphere in their working space. A right climate is unavoidable if we want to keep the team's performance high.

A warm and pleasant ambiance in the workplace is not enough without an intentional plan to provide psychologically friendly interaction and a good atmosphere to practice exploration and exploitation of ambidextrous leadership. It is our homework as leaders to create a good climate and provide ambidextrous friendly circumstances for every start-up team.

The result of the study leads to an understanding of important aspects of keeping good team performance in a start-up team. A comprehensive approach is needed to equip team leaders with suitable behavior for Team Start-ups. A research-based leadership training module can be developed not only for capacity building but for fostering creativity among business people and professionals since most training modules do not have deep theoretical and empirical roots.

#### Research limitation

In terms of area of study, this research has limitations. First, it focuses on the start-up teams in several big cities in Indonesia, such as Surabaya, Jakarta, and Bandung. Various cities in Indonesia or other countries may give different results due to the cultural context. The other limitation is the type of start-up business. This study only covers seven types of business such as tourism (including culinary business), personal development, fashion, design and marketing, trading, technology-based, and social entrepreneurship with the same treatment. We believe that Team Start-ups with other business types will differ in response to leadership behavior. This study exercises how Team Creativity emerges in the team context without external interruption such as investor intervention, although we believe that investors can be the final decision maker in creativity.

#### Recommendation for further research

This study leaves a lot of room for further research in the field of the team aspect and its derivatives such as team anxiety, team culture, and team resilience. In the era of millennial workers, the issue of the team is important. While millennials are often considered individualistic, they can become good team players eventually. Furthermore, this study opens the door for Indonesian and other countries' ethnic and cultural leadership studies. A study of team leadership aspects in multigroup, longitudinal, and experimental research based on geographical, gender, technology, and team composition is recommended. The research methodology may be extended to longitudinal and experimental both randomized and non-randomized subject research is needed as further research on leadership behavior and its influence on innovation and creativity (Uy et al., 2021).

## Conclusions

This study begins with a single question what are the mediators for a team leader to foster Team Creativity in Team Start-up? Starting with a theoretical study of previous research in leadership behaviors and start-ups, we propose two types of leadership, Transformational Leadership and Servant Leadership, and two potential mediators, Team Ambidexterity and Team Climate. The sum of evidence presented in the data analyses confirms that Team Ambidexterity and Team Climate are good moderators for Team Creativity.

This simple question has far-reached implications for articulating leadership theory and its application in Team Start-ups. Good team leaders are imperative for Team Start-ups to maintain their performance through creativity. Both vertical leadership and horizontal leadership types play a significant role in embracing the creativity and innovation process in start-ups. Team leaders should equip themselves to keep the performance of the team. A start-up's success depends not only on the team but also on the team leader.

We believe that the mediator's role is significant to keep the start-up's performance well. Our mediation analysis shows that building a better Team Climate will enhance the team member to be more creative. Work climate in the young generation is more important than other aspects in the co-working space. Creating a warm and conducive environment in a start-up team is unavoidable to keep the business running. Second, to Team Climate, Team Ambidexterity is good to empower the team member with creativity. Therefore, the combination of the exploration and exploitation process is necessary to optimize all team members' competence and talent.

The growing start-up business shifts the leadership struggle from a big organization to a smaller team context. A deeper understanding of team leadership and its mediator and the moderator is essential for theoretical and practical start-up development.

## Data availability statement

The original contributions presented in the study are included in the article/supplementary material, further inquiries can be directed to the corresponding author/s.

## Ethics statement

Ethical review and approval was not required for the study on human participants in accordance with the local legislation and institutional requirements. Written informed consent from the [patients/ participants OR patients/participants legal guardian/next of kin] was not required to participate in this study in accordance with the national legislation and the institutional requirements.

## Author contributions

TA initiates the research model before it is discussed and improved by all authors and provides the initial study of the theoretical background. AI and LP responsible for the sample collection. All authors did the data analysis and the discussion and conclusion of the research. All authors contributed to the article and approved the submitted version.

## Funding

This research program was funded by the Ministry of Education, Culture, Research, and Technology, the Republic of Indonesia, under the research grant no. 005/UC-LPPM/PT-L/V/2022 of the Directorate General of Higher Education.

## Conflict of interest

The authors declare that the research was conducted in the absence of any commercial or financial relationships that could be construed as a potential conflict of interest.

## Publisher's note

All claims expressed in this article are solely those of the authors and do not necessarily represent those of their affiliated organizations, or those of the publisher, the editors and the reviewers. Any product that may be evaluated in this article, or claim that may be made by its manufacturer, is not guaranteed or endorsed by the publisher.
